# Comparative genomic analysis of bacteriophages specific to the channel catfish pathogen *Edwardsiella ictaluri*

**DOI:** 10.1186/1743-422X-8-6

**Published:** 2011-01-07

**Authors:** Abel Carrias, Timothy J Welch, Geoffrey C Waldbieser, David A Mead, Jeffery S Terhune, Mark R Liles

**Affiliations:** 1 Department of Fisheries and Allied Aquaculture, Auburn University, USA; 2 National Center for Cool and Cold Water Aquaculture, Agricultural Research Service, USDA, Kearneysville, WV 25430, USA; 3 USDA, ARS, Catfish Genetics Research Unit, Thad Cochran National Warm Water Aquaculture Center, Stoneville, Mississippi, USA; 4 Lucigen Corporation, Middleton, WI, USA; 5 Department of Biological Sciences, Auburn University, USA

## Abstract

**Background:**

The bacterial pathogen *Edwardsiella ictaluri *is a primary cause of mortality in channel catfish raised commercially in aquaculture farms. Additional treatment and diagnostic regimes are needed for this enteric pathogen, motivating the discovery and characterization of bacteriophages specific to *E. ictaluri*.

**Results:**

The genomes of three *Edwardsiella ictaluri*-specific bacteriophages isolated from geographically distant aquaculture ponds, at different times, were sequenced and analyzed. The genomes for phages eiAU, eiDWF, and eiMSLS are 42.80 kbp, 42.12 kbp, and 42.69 kbp, respectively, and are greater than 95% identical to each other at the nucleotide level. Nucleotide differences were mostly observed in non-coding regions and in structural proteins, with significant variability in the sequences of putative tail fiber proteins. The genome organization of these phages exhibit a pattern shared by other *Siphoviridae*.

**Conclusions:**

These *E. ictaluri*-specific phage genomes reveal considerable conservation of genomic architecture and sequence identity, even with considerable temporal and spatial divergence in their isolation. Their genomic homogeneity is similarly observed among *E. ictaluri *bacterial isolates. The genomic analysis of these phages supports the conclusion that these are virulent phages, lacking the capacity for lysogeny or expression of virulence genes. This study contributes to our knowledge of phage genomic diversity and facilitates studies on the diagnostic and therapeutic applications of these phages.

## Background

Here we report the complete nucleotide sequence and annotation of the genomes of three bacteriophages specific to the gram negative bacterial pathogen *Edwardsiella ictaluri*, the causative agent of enteric septicemia of catfish (ESC). ESC is a primary cause of mortality in catfish farms with annual direct losses in the range of $40-60 million dollars in the U.S. [1]. Economic losses coupled with limited available treatment options for controlling ESC, and concerns regarding the development of resistance to antibiotics used in aquaculture warranted efforts to identify biological control agents that are antagonistic to *E. ictaluri *(e.g., bacteriophage and bacteria). In addition, the multiple days necessary to obtain a diagnostic result for *E. ictaluri *via biochemical tests was a motivation to identify phage that could serve as specific, rapid, and inexpensive typing agents for ESC disease isolates.

The idea of using phage as antimicrobial agents to treat bacterial infections in agriculture or aquaculture is not a new proposition [2]; however, there is now a better understanding of phage biology and genetics, and with it a better understanding of their potential and their limitations as biological control agents [3]. The most serious obstacles to successful use of phage therapy include the development of phage resistance by host bacteria, the capacity of some temperate phages to transduce virulence factors (i.e., lysogenic conversion), the possible degradation or elimination of phages by gastrointestinal pH or proteolytic activity within a fish, and the possible immune system clearance of administered phage. Potentially viable solutions are available to counter each of these concerns, including the use of multiple phages at concentrations selected to reduce the development of phage-resistant bacterial populations [4], identifying phage variants adapted to minimize GI tract and/or immune clearance [5], and by selecting bacteriophages as therapeutic agents that are well characterized at a genomic level, with no potential for inducing lysogenic conversion [2,3,6].

Two unique *E. ictaluri*-specific phages ϕeiAU (eiAU) and ϕeiDWF (eiDWF) were isolated from aquaculture ponds with a history of ESC [7]. Phage eiAU was isolated in 1985 at Auburn University and phage eiDWF was recently isolated in 2006 in western Alabama. An additional *E. ictaluri*-specific bacteriophage ϕeiMSLS (eiMSLS) was isolated directly from culture water from a commercial catfish aquaculture pond in Washington County, MS in 2004 (Timothy Welch, USDA National Center for Cool and Cold Water Aquaculture, WV personal communication). The isolation of each of these bacteriophages was accomplished by concentrating viruses from pond water samples by ultrafiltration and enriching for *E. ictaluri*-specific bacteriophages via enrichment in log-phase bacterial broth cultures. These three bacteriophages were classified initially within the family *Siphoviridae *due to their long, non-contractile tails, but their phylogenetic affiliation could not be assessed in the absence of phage genome sequence analysis [8-10]. To date no other bacteriophage morphotypes have been observed to infect *E. ictaluri *from pond water enrichment experiments. A genomic analysis of these three phages was initiated to examine the potential of these three bacteriophages for lysogeny, to ensure they did not harbor virulence or toxin genes and to better understand the genetic basis of their host specificity [7]. This study represents the first genomic analysis of bacteriophages specific to *Edwardsiella ictaluri*, and will expand scientific understanding of phage biology, and genomic information [11].

## Results and Discussion

### Genome characteristics

Total sequence coverage for the eiMSLS assembly was 9.8X, while coverage for the eiAU and eiDWF assemblies exceeded 30X. The genomes of phages eiAU, eiDWF, and eiMSLS are 42.80 kbp, 42.12 kbp, and 42.69 kbp, respectively. The % GC content is 55.37%, 55.54%, and 55.77% for phage eiAU, eiDWF, and eiMSLS, respectively, and is similar to the 57% GC content of host *E. ictaluri *genome reference strain (GenBank accession NC 012779). No tRNA genes were detected in the genome of any of the three phages. This is unlike several members of the *Siphoviridae *family that carry tRNA genes [12].

### Open Reading Frame (ORF) analysis

A total of 54 ORFs were predicted for phage eiAU (Table 1), while 52 ORFs were predicted for eiDWF and 52 ORFs for eiMSLS. Based on sequence similarity (E value < 0.001), 40 out of 54 (74%), 37 out of 52 (71%) and 36 out of 52 (69%) of the ORFs for phages eiAU, eiDWF, and eiMSLS, respectively, share significant sequence similarity to known protein sequences contained in the GenBank nr/nt database (Table 1). Of the ORFs with significant sequence similarity to sequences in GenBank, putative functions could only be assigned to 21 out of 40 (53%), 21 out of 37 (57%) and 20 out of 36 (56%) for phages eiAU, eiDWF, and eiMSLS, respectively. Positions, sizes, sequence homologies and putative functions for each predicted ORF are presented in Table 1.

**Table 1:** Predicted ORFs for eiAU, eiDWF, and eiMSLS, and the most similar BLAST hits for each of the phage ORFs

ϕeiAU ORF/Strand	Position		Size		Putative function [Nearest neighbor]	Accession #	Best match E value/% aa identity	Presence in	
	Start	Stop	bp	aa				ϕeiDWF	ϕ MSLS
1/-	220	459	240	79	None			[+]	[+]
2/+	458	925	468	155	DNA Repair ATPase [*Salmonella *phage]	YP_003090241.1	1E-49/67	[+]	[+]
3/+	922	1260	339	112	None			[+]	[+]
4/+	1319	2668	1350	449	helicase [*Enterobacteria *phage]	YP_002720041.1	0.0/70	[+]	[+]
5/+	3035	3211	177	58	None			[-]	[-]
6/+	3239	4126	888	295	phage methyltransferase [*Edwardsiella tarda*]	ZP_06713110.1	4E-98/69	[+]	[+]
7/+	4126	4836	711	236	N-6-adenine-methyltransferase [*Escherichia coli*]	YP_003041971.1	3e-18/45	[-]	[-]
8/+	5164	5526	363	120	None			[+]	[+]
9/+	5523	5804	282	93	None			[+]	[+]
10/-	6073	5816	258	85	None			[+]	[-]
11/-	6581	6060	522	173	hypothetical protein [Phage PY100]	CAJ28429.1	8E-20/38	[+]	[+]
12/-	6869	6603	267	88	None			[+]	[+]
13/-	7721	7020	702	233	hypothetical protein [Phage PY100]	CAJ28427.1	2E-09/36	[+]	[+]
14/-	8175	7822	354	117	phage tail assembly chaperone gp38 [*Enterobacter *sp.]	YP_001178193.1	9e-11/53	[+]	[-]
15/-	9179	8172	1008	335	tail fiber protein [*Enterobacteria *phage]	NP_037718.1	2e-10/38	[+]	[+]
16/-	12809	9198	3612	1203	phage host specificity protein [*Yersinia kristensenii*]	ZP_04623740.1	0.0/42	[+]	[+]
17/-	13333	12809	524	174	phage tail assembly protein [*Yersinia enterocolitica *phage]	YP_001006526.1	8E-50/59	[+]	[+]
18/-	14112	13393	720	239	phage minor tail protein [*Enterobacteria *phage]	YP_002720062.1	4E-56/48	[+]	[+]
19/-	14887	14117	771	256	phage minor tail protein L [*Yersinia pseudotuberculosis*]	YP_001721823.1	5E-66/51	[+]	[+]
20/-	15228	14884	345	114	phage minor tail protein M [*Enterobacteria *phage phi80]	CBH95068.1	1E-12/39	[+]	[+]
21/-	17990	15288	2703	900	phage tail tape measure protein [*Enterobacteria *phage]	YP_002720065.1	8E-126/38	[+]	[+]
22/-	19188	18862	327	108	gp16 [*Sodalis *phage SO-1]	YP_003344951.1	5E-20/48	[+]	[+]
23/-	19523	19167	357	118	gp15 [*Sodalis *phage SO-1]	YP_003344950.1	9E-16/38	[+]	[+]
24/-	20305	19703	603	200	putative major tail protein [*Enterobacteria *phage]	YP_002720068.1	2E-54/58	[+]	[+]
25/-	20766	20338	429	142	gp13 [*Sodalis *phage SO-1]	YP_003344948.1	1E-08/38	[+]	[+]
26/-	21395	20763	633	210	gp12 [*Sodalis *phage SO-1]	YP_003344947.1	6E-53/55	[+]	[+]
27/-	21748	21392	357	118	phage structural protein [*Enterobacteria *phage]	YP_002720071.1	9E-23/48	[+]	[+]
28/-	21884	21729	156	51	None			[+]	[+]
29/-	22387	21887	501	166	hypothetical protein EpSSL_gp33 [*Enterobacteria *phage]	YP_002720072.1	2E-22/43	[+]	[+]
30/-	23550	22450	1065	353	phage structural protein [*Enterobacteria *phage]	YP_002720073.1	1E-65/59	[+]	[+]
31/-	24306	23638	669	222	hypothetical protein EpSSL_gp36 [*Enterobacteria *phage]	YP_002720075.1	1E-39/50	[+]	[+]
32/-	25520	24393	1128	375	phage head morphogenesis protein [*Enterobacteria *phage]	YP_002720086.1	2E-123/58	[+]	[+]
33/-	26964	25504	1461	486	phage structural protein [*Enterobacteria *phage]	YP_002720085.1	1E-153/57	[+]	[+]
34/-	28358	26976	1383	460	phage terminase large subunit [*Enterobacteria *phage]	YP_002720084.1	4E-162/64	[+]	[+]
35/-	28855	28358	498	165	gp1 [*Sodalis *phage]	YP_003344936.1	2E-24/48	[+]	[+]
36/-	29356	29090	267	88	endolysin [*Yersenia *Phage PY100]	CAJ28446.1	7E-14/48	[+]	[+]
37/-	29775	29500	276	91	prophage Lp2 protein 33 [*Streptococcus pneumonia*]	ZP_01821446.1	2E-09/45	[+]	[+]
38/-	30311	29826	486	161	putative lysis accessory protein [*Escherichia *phage]	YP_512284.1	1E-10/39	[+]	[+]
39/-	30559	30308	381	127	Putative holin [Burkholderia multivorans CGD1]	ZP_03586913.1	5E-05/30	[+]	[+]
40/-	30996	30775	222	73	None			[+]	[+]
41/-	31670	31026	645	214	None			[+]	[+]
42/-	32769	32128	642	213	Conserved phage protein [*Enterobacteria *phage]	ADE87955.1	2E-27/37	[+]	[+]
43/-	33112	32882	231	76	None			[+]	[+]
44/-	35397	33988	1410	469	phage replicative helicase/primease [*Enterobacteria *phage]	YP_002720055.1	7E-114/58	[+]	[+]
45/+	35764	36093	330	109	None			[+]	[+]
46/+	36115	36282	168	55	None			[+]	[+]
47/-	36455	36339	117	38	None			[+]	[+]
48/+	36834	37277	444	147	gp46 [*Sodalis *phage]	YP_003344981.1	5E-04/36	[-]	[+]
49/+	37326	37862	537	178	gp27 [*Sodalis *phage]	YP_003344962.1	5E-04/40	[+]	[+]
50/+	37865	38098	234	77	None			[-]	[+]
51/+	38101	39360	1194	396	gp43 [*Sodalis *phage]	YP_003344978.1	9E-49/50	[+]	[+]
52/+	39455	40192	738	245	gp41 [*Sodalis *phage]	YP_003344976.1	4E-56/64	[+]	[+]
53/+	40252	42459	2208	735	DNA polymerase I [*Enterobacteria *phage]	YP_002720046.1	0.0/64	[+]	[+]
54/+	42470	42748	279	92	gp36 [*Sodalis *phage SO-1]	YP_003344971.1	1E-22/60	[+]	[+]

The genome of phage eiAU contains several overlapping predicted ORFs, which can be an indication of translational coupling or programmed translational frameshifts [13]. Twelve possible sequence frameshifts were predicted in the eiAU genome sequence. Interestingly, one of these frameshifts is conserved in tail assembly genes of dsDNA phages [14]. In dsDNA phage genomes the order of the tail genes is highly conserved, most notably the major tail protein is always encoded upstream of the gene encoding the tape measure protein [14]. Between these two genes, two overlapping ORFs are commonly found that have a translational frameshift [15]. A similar organization of tail genes is observed in phage eiAU, in which two ORFs (22 and 23) lie between the putative phage tape tail measure protein gene (ORF21) and the major tail protein (ORF24) (Table 1). Similarly, phage eiAU contains a frameshift in the two overlapping ORFs between the phage tail measure and the major tail protein. In other phages both of these proteins are required for tail assembly even though they are not part of the mature tail structure [14].

### Overall Genome Organization and Comparison

**Figure 1:**
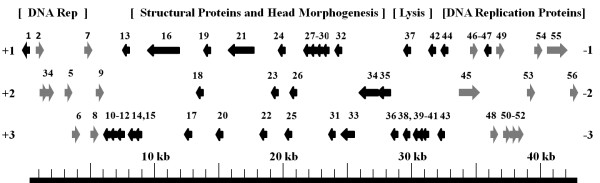
**Schematic representation of the genome sequence of bacteriophage eiAU showing its overall genomic organization**. The ORFs are numbered consecutively (see Table 1) and are represented by arrows based on the direction of transcription. The numbers +1, +2, +3 represent corresponding reading frames.

**Figure 2:**
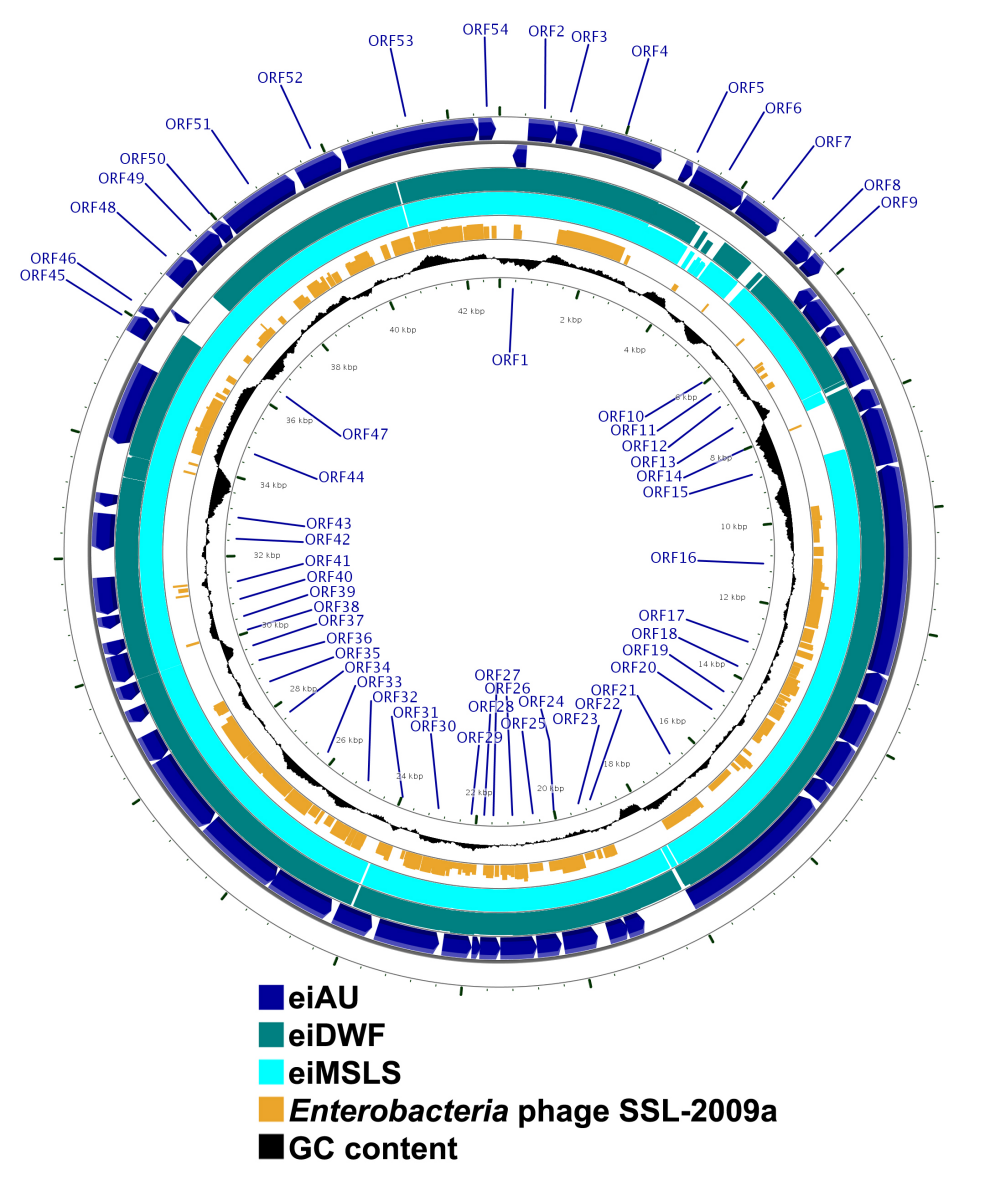
**Circular representation depicting the genomic organization of eiAU (two outermost circles, dark blue, showing each predicted ORF and its direction of transcription) and a tBLASTx comparison with the genomes of eiDWF (third circle from outside, green), eiMSLS (fourth circle from outside, light blue), and *Enterobacteria *phage SSL-2009a (fifth circle from outside, orange)**. The degree of sequence similarity to eiAU is proportional to the height of the bars in each frame. The %G+C content of eiAU is also depicted (sixth circle from outside, black). This map was created using the CGView server (Grant and Stothard, 2008).

A schematic representation of one of these phages (eiAU) shows that ORFs in these three phages are organized into two groups; early genes (DNA replication) that are encoded on one strand and the late genes (head, tail, and lysis) that are encoded on the complementary strand (Figure 1). Whole genome comparisons revealed that phages eiAU, eiDWF, and eiMSLS have conserved synteny (Figure 1 and Figure 2). The overall genetic organization of the eiAU, eiDWF, and eiMSLS genomes, typically consisting of "DNA packaging-head-tail-tail fiber-lysis/lysogeny-DNA replication-transcriptional regulation" modules is shared by many phage within the *Siphoviridae *family [16].

Multiple sequence alignment analysis revealed that the eiAU, eiDWF, and eiMSLS genomes are >95% identical at the nucleotide level (Figure 2). Similarly, a high degree of sequence similarity has been observed in the genomes of recently sequence bacteriophages that infect *Campylobacter *[17], *Eschericia coli *[18], and also many *Mycobacterium *spp. [19]. The high similarity of some phage genomes that infect a single host species suggests that certain phage lineages may be stable over time and over distant geographic areas [17]. This observation may likely be clarified once additional genome sequences of phages infecting a common host such as *E. ictaluri *become available.

### Comparison of head morphogenesis and structural proteins

Genome sequencing of tailed phages and prophages has revealed a common genetic organization of the genes encoding head morphogenesis and head structural proteins. These gene systems are typically organized as follows: 'terminase - portal - protease - scaffold - major head shell (coat) protein - head/tail-joining proteins - tail shaft protein - tape measure protein - tail tip/base plate proteins - tail fiber' (listed in the order of transcription) [20]. Phages eiAU, eiDWF, and eiMSLS follow a similar organization of genes encoding head morphogenesis and structural proteins, although the direction is reversed in relation to their order of transcription (Figure 1 and Table 1).

The module containing head morphogenesis and tail structure proteins in phage eiAU is the largest module, and is predicted to contain 22 ORFs (ORF14-ORF35). The consecutive ORFs 14 to 32 have significant sequence similarity with phage head morphogenesis and structural proteins, with putative function in tail assembly (ORFs 14, 17, and 18), tail fiber protein (ORF 15), phage host specificity (ORF 16), minor tail proteins (ORFs 19-21), major tail proteins (ORFs 24 and 25), major capsid proteins (ORF 29), structural proteins (ORFs 27, 30 and 33), and a phage head morphogenesis protein (ORF32) (Table 1). ORFs 28, 26, 23, and 22 could not be linked to a putative function based on BLAST search or any other similarity searches. However, all of these ORFs with the exception of ORF28 have sequence similarity to proteins identified within other phage genomes (Table 1). The protein products of ORF34 and ORF35 may encode large and small terminase subunits, respectively. ORF34 is predicted to encode the terminase large subunit. The top BLAST hit for ORF35 is the protein Gp1 encoded by Sodalis phage SO-1; however, it is possible that ORF 35 encodes a small terminase subunit as there is limited sequence similarity to a putative terminase small subunit from *Listonella *phage phiHSIC. This indicates that these *E. ictaluri *phages, similarly to most dsDNA viruses, use a DNA packaging motor consisting of two nonstructural proteins (the large and small terminase subunits) encoded by adjacent genes [21]. Most known terminase enzymes have a small subunit that specifically binds the viral DNA and the large subunit with endonuclease activity for DNA cleavage and an ATPase activity that powers DNA packaging [22,23].

No hit for a portal protein or for a protease was obtained either by BLAST or by HmmPfam searches. ORF33 is the most likely candidate for a portal protein based on the observation that the portal protein is generally located immediately downstream of the terminase gene [13].

### Lytic Cassette

The lytic cassette of phage eiAU is predicted to be encoded by ORFs 36-39. ORF36 encodes a predicted endolysin, and a putative holin protein is encoded by ORF39. All dsDNA phages studied to date use two enzymes to lyse their host, an endolysin which degrades cell wall peptidoglycan and a holin which permeabilizes the cell membrane [21]. These two proteins work in conjunction to destroy the cell wall of bacteria and subsequently lyse the cell [24]. These components of a host lysis cassette are each present in the genome of phages eiAU, eiDWF, and eiMSLS including a putative Rz lysis accessory protein encoded by ORF38 (Table 1.). The RZ protein is predicted to be a type II integral membrane protein and its function, although not fully understood, may be required for host cell lysis only in a medium containing an excess of divalent cations [25]. Phage endolysins have been linked to five enzymatic activities, including an N-acetyl muramidase or "true lysosyme", the lytic transglycosylases, the N-acetylmuramoyl-L-alanine amidases, the endo- β-*N*-acetylglucosaminidases, and the endopeptidases [26]. Secondary structure analysis predicts that the endolysin of eiAU is a member of the N-acetylmuramoyl-L-alanine amidases class of endolysins.

### DNA replication proteins

ORFs with significant sequence similarity to proteins involved in DNA replication were identified in all three *E. ictaluri*-specific phage genomes. ORF44 is predicted to encode a phage replicative helicase/primease. Several phages use separate primase and helicase proteins while others use a multifunctional protein (primase/helicase) possessing both activities [13]. The helicase/primase protein works in DNA replication by unwinding double stranded DNA into single stranded DNA [27]. No predicted function could be assigned to ORFs45 and 46. Also, no predicted function could be assigned to ORF47; however, a search for secondary structures within the predicted ORF47 amino acid sequence detected a helix-hairpin-helix DNA binding motif. Additionally, no putative function could be assigned to ORF48, ORF49, or ORF50. ORF51 had as one of its top BLAST hits an isoprenylcysteine carboxyl methyltransferase known to function in methylating isoprenylated amino acids [28]. ORF52 is predicted to encode a protein similar to gp41 of *Sodalis *phage SO-1, but no putative function could be assigned. ORF53 is predicted to encode DNA polymerase I. Secondary structure analysis suggested that the DNA polymerase encoded by ORF53 contains a domain that is responsible for the 3'-5' exonuclease proof-reading activity of *E. coli *DNA polymerase I and other enzymes, and catalyses the hydrolysis of unpaired or mismatched nucleotides. The protein encoded by ORF54 is predicted to have a VUR-NUC domain, which are associated with members of the PD-(D/E) XK nuclease superfamily such as type III restriction modification enzymes. ORF2 is predicted to encode a DNA repair ATPase. A search for secondary structures within the ORF2 predicted amino acid sequence revealed a HNH endonuclease. No putative function could be assigned to ORF3. ORF4 is predicted to encode a helicase protein belonging to the SNF2 family, commonly found in proteins involved in a variety of processes including transcription regulation, DNA repair, DNA recombination, and chromatin unwinding [29]. ORF6 is predicted to encode a phage methyltransferase. Secondary structure analysis revealed that the methyltransferase predicted to be encoded by ORF6 is a C-5 cytosine-specific DNA methylase which in bacteria is a component of restriction-modification systems. Also, Mg^+ ^and ATP binding sites were detected in the predicted protein product of ORF6. ORF7 is predicted to encode a DNA N-6-adenine-methyltransferase within a family of methyltransferase found in bacteria and phage that has site specific DNA methyltransferase activity [30].

No ORF encoding an RNA polymerase was detected in any of the phages suggesting that these phages rely on the host RNA polymerase to transcribe their genes. This is further corroborated by the observation that no phage-encoded transcription factor was detected in the genome of these phages.

### Comparison of ORFs among phages eiAU, eiDWF, and eiMSLS

The three phage genomes revealed extensive homology and limited variability in their gene sequence (Figure 2). The percent identity and percent similarity of each ORF within the three phage genomes (data not shown) revealed that differences exist mainly in predicted ORFs that have no significant sequence similarity to sequences in GenBank database and also to ORFs encoding structural proteins (primarily the tail fiber genes). ORF14 (117 AA) is predicted to encode a phage tail fiber assembly protein/tail assembly chaperone, and in eiAU and eiDWF it is 100% identical, yet it is not present in eiMSLS. ORF15 (335 AA) is predicted to encode a tail fiber protein and is present in all three phages, with 100% identity in eiAU and eiDWF, however, it only has 58% identity to its counterpart in eiMSLS. ORF21 (900 AA) is predicted to encode a phage tail tape measure protein and is present in all three phages at approximately 95% identity at the amino acid level. ORF23 (118 AA) is predicted to encode a protein homologous to gp15 [*Sodalis *phage SO-1] which is a structural protein that plays a role in cell membrane penetration. This ORF is present in all three phages with 83% identity at the amino acid level. ORF24 (200 AA) is predicted to encode a major tail protein and is present in all three phages, with 100% identity between eiDWF and eiMSLS, and with only 90% identity between those two phage and the ORF counterpart in eiAU. Sequence differences in these structural proteins may help explain the differences observed in the efficiency of these phages to form plaques on various *E. ictaluri *strains [7]. Most of the structural proteins described above are expected to be involved in phage infectivity such as adsorption of the phage to the bacterial cell (ORFs 14 and 15), phage tail length (ORF21), and cell membrane penetration (ORF23).

Differences were also observed in the ORFs encoding the putative methyltransferases. In phage eiAU, ORF6 and ORF7 are predicted to encode a phage methyltransferase and a DNA N-6-adenine-methyltransferase respectively, while in phage eiDWF and eiMSLS only one larger ORF encoding a phage methyltransferase was predicted. Similarly, two methyltransferases are present in the genomes of one of two highly similar *Campylobacter *phages [17]. The authors suggest that the two methyltransferases may enable the phage to avoid DNA restriction in some strains through DNA methylation. This may help explain the differences observed in host range for the *Campylobacter *phages [17] as well as differences observed in host specificity of the *E. ictaluri *phages [7]. Hence, these methyltransferases may likely be involved in DNA methylation as a means of avoiding the restriction endonuclease (s) of *E. ictaluri*.

### Classification of phages eiAU, eiDWF, and eiMSLS

The majority of the top BLAST hits for these phage genomes are to proteins belonging to lytic phages, including *Yersinia *phage PY100, *Salmonella *phage c341, and *Enterobacteria *phage HK97 (Table 1.). All of the components of a phage lysis cassette (endolysin, holin, and a lysis accessory protein) were detected in these phages and no sequence similarity to lysogenic phages or to any component that is associated with lysogeny such as integrase/recombination associated enzymes, repressor proteins, and anti-repressor proteins [31] were detected. These data along with results documenting the lytic capabilities of these phages [7] all indicate that these phages lack mechanisms for integration into the DNA of their host and that they are virulent phages without the capacity for lysogeny. Additionally, none of the predicted proteins have similarities to known bacterial pathogenicity factors. These observations indicate that these phages lack any lysogenic or bacterial virulence-inducing capacity that would preclude their potential use as therapeutic agents.

Taxonomic classification of these *E. ictaluri*-specific phages must rely upon a synthesis of morphological and genomic information, considering that phage evolution has been profoundly directed by lateral gene transfer [32], and that a rational hierarchical system of phage classification should be based on the degree of DNA and protein sequence identity for multiple genetic loci [33]. Gene modules that have been proposed for using as basis of a phage taxonomy system include the DNA packaging-head gene cluster, the structural gene architecture, and phage tail genes (excluding the tail fiber genes) [16].

A comparison of phage eiAU to *Enterobacteria *phage SSL-2009a was conducted due to the large number of significant BLAST hits between ORFs in the *E. ictaluri *phage genomes and those respective ORFs within the genome of phage SSL-2009a, which are on average 34.1% identical at the nucleotide level. A comparative genomic analysis between the genome of phage eiAU and that of phage SSL-2009a revealed that genome regions encoding many putative structural and replication proteins are shared by both phages (Figure 2). The predicted gene products with sequence similarity between the eiAU and SSL-2009a phage genomes include the putative minor tail proteins/tail tape measure, major tail proteins, major capsid proteins, head morphogenesis, phage terminase small subunit, and the phage terminase large subunit. Interestingly, other structural proteins including the host specificity proteins, the tail assembly proteins, and particularly the tail fiber/baseplate protein which has been recommended for exclusion in any sequence based phage taxonomy scheme [33] are not shared between the two genomes.

### Phylogeny based on multiple genetic loci

**Figure 3:**
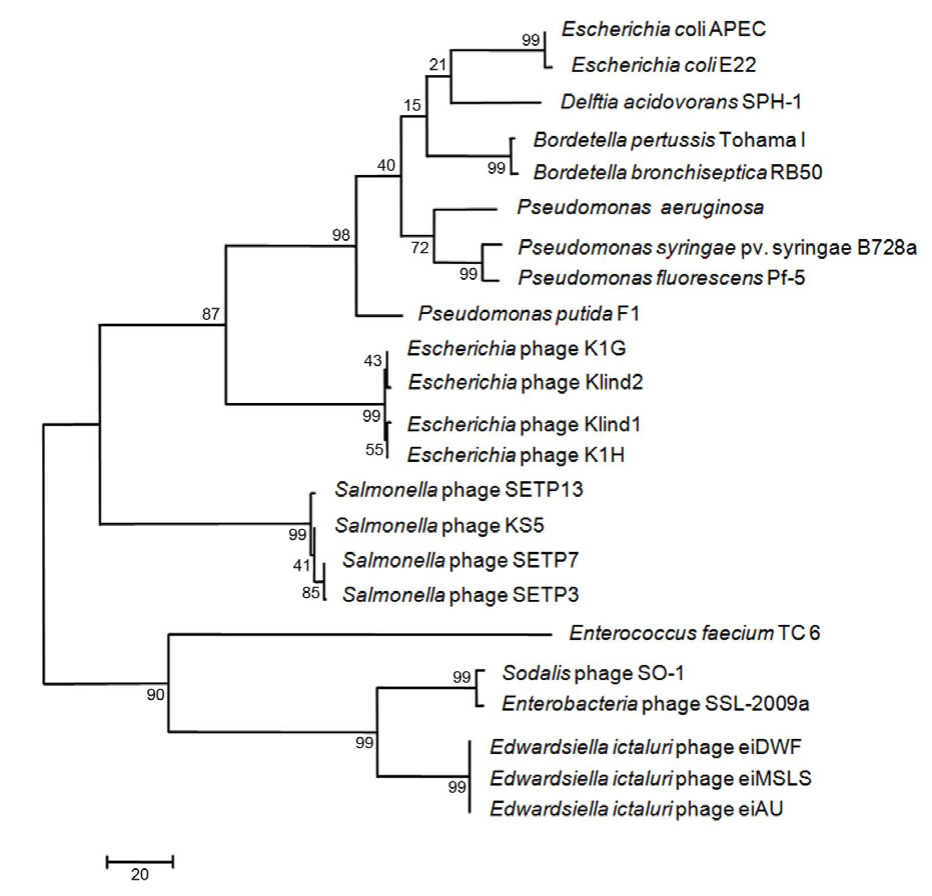
**Rooted maximum parsimony tree based on the aligned amino acid sequences of the large terminase subunit gene of phage eiAU and 25 other large terminase genes from diverse phage genomes**. The numbers at the nodes represent bootstrap values based on 1,000 resamplings.

**Figure 4:**
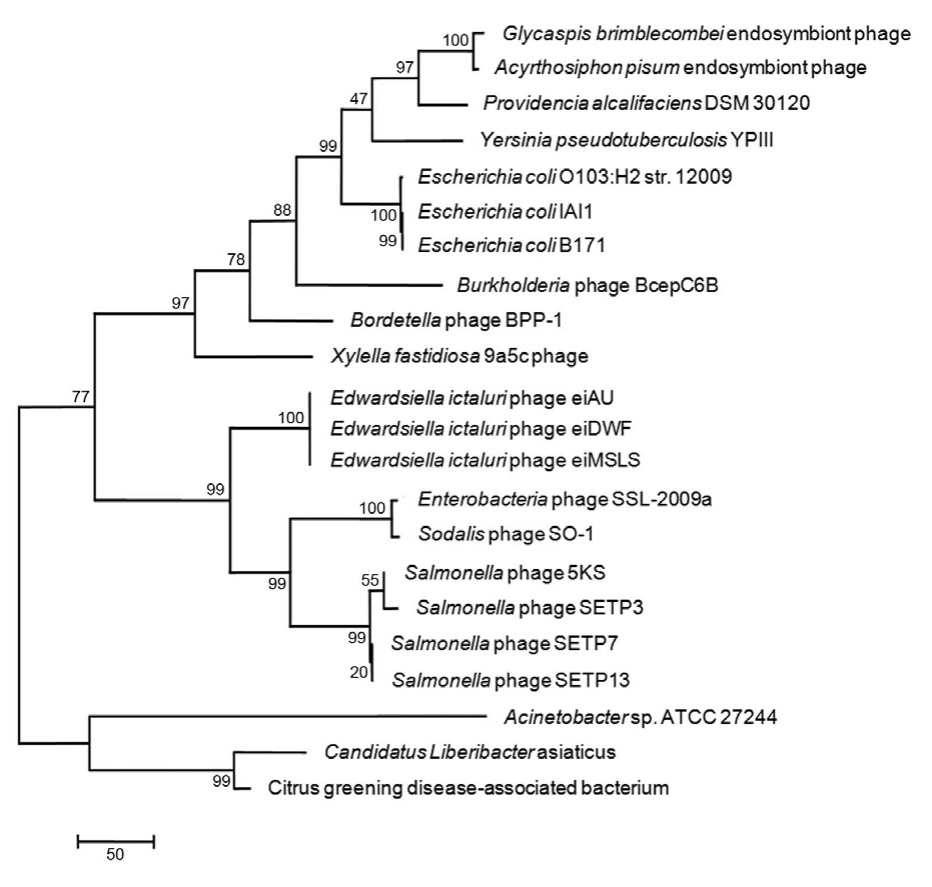
**Rooted maximum parsimony tree based on the aligned amino acid sequences of the DNA polymerase subunit gene of phage eiAU and 33 other DNA Polymerases from diverse phage genomes**. The numbers at the nodes represent bootstrap values based on 1,000 resamplings.

The genetic conservation observed in the structural proteins between phage eiAU and *Enterobacteria *phage SSL-2009a led us to further investigate the relatedness of these *E. ictaluri *phages and other enterobacteria phage, based on specific phage genetic loci. The amino acid sequences of one of the conserved structural proteins (large terminase subunit) as well as one of the non structural proteins (DNA polymerase I) were chosen for phylogenetic analysis. The large terminase subunit which is a structural protein is along with the portal protein considered the most universally conserved gene sequence in phages [20], hence they are good options to aid in phage classification. Phylogenetic analysis based on the large terminase subunit amino acid sequence (Figure 3) and the DNA polymerase I amino acid sequence (Figure 4) of eiAU reveal that phages eiAU, eiDWS, and eiMSLS were most similar to phage that infect other enterobacteria (*Enterobacteria *phage SSL-2009a) and *Sodalis glossinidius *(*Sodalis *phage SO-1). These two phages are dsDNA viruses belonging to the Caudovirales order, one being a *Siphoviridae *(*Sodalis *phage SO-1) (NCBI accession # NC_013600) and the other an unclassified member of the Caudovirales (*Enterobacteria *phage SSL-2009a) (NCBI accession # NC_012223). The overall genomic organization of the three new phages is shared by many members of the *Siphoviridae *family of phages sequenced to date [16], and is supported by the previously described morphology of these phages [7].

## Conclusion

This is the first genomic analysis of bacteriophages that infect the bacterial pathogen *E. ictaluri*. Phylogenetic analysis of multiple phage gene products suggests that these phages are similar to those that infect other *Enterobacteria *hosts. The bioinformatic analysis of the genomes of these three *E. ictaluri*-specific bacteriophages corroborate previously published data that indicates that these bacteriophages are lytic, and lack any mechanism for lysogenic conversion of their host. Additionally, none of the predicted proteins have similarities to known bacterial pathogenicity factors or to toxin genes. Even though these three bacteriophages were isolated in different geographic locations within the natural range of catfish over twenty years apart, they are remarkably similar to each other at a genomic level. This genomic analysis suggests that these phages are members of a lineage that is highly stable over time and geographic regions. The information obtained from the analyses of these bacteriophage genomes will facilitate their diagnostic and therapeutic applications.

## Methods

### Bacteriophages and bacterial strains

Phages ϕeiAU and ϕeiDWF used in the study were originally isolated and characterized at Auburn University [7]. Phage ϕMSLS was isolated from an aquaculture pond water sample on a lawn of *E. ictaluri *strain I49 (Thad Cocharan National Warmwater Aquaculture Center, Aquatic Diagnostic Lab), and clear plaques were doubly purified on an *E. ictaluri *host. Host bacterial isolate *E. ictaluri *strain 219 was obtained from the Southeastern Cooperative Fish Disease Laboratory at Auburn University. *E. ictaluri *strains were grown on Brain Heart Infusion (BHI) medium and cryopreserved in BHI containing 10% glycerol at -80°C. In each experiment bacterial strains were grown from the original glycerol stock to maintain low passage number, virulent *E. ictaluri *cultures.

### Isolation of phage DNA

Phages eiAU, eiDWF, and eiMSLS were propagated on *E. ictaluri *strain 219 using a standard soft agar overlay method [34]. Phages were harvested by flooding plates with 5 mL SM buffer (100 mM NaCl, 8 mM MgSO_4_·7H_2_O, 50 mM Tris-Cl (1 M, pH 7.5), and 0.002% (w/v) of 2% Gelatin), incubating at 30°C while shaking for 6 h, and then collecting the buffer-phage solution. Collected phage suspensions were treated for 10 min with 1% (v/v) chloroform to lyse bacterial cells, subjected to centrifugation at 3,600× g for 25 min, and then filtered through a 0.22 μm filter to remove cell debris. Phage solutions were purified over a cesium chloride gradient and concentrated by precipitation with polyethylene glycol 8000. Concentrated phage particles were resuspended in 200 μl SM buffer. Free nucleic acids from lysed bacterial host cells were degraded with 250 units of benzonase endonuclease for 2 h at 37°C, after which the benzonase was inhibited by the addition of 10 mM EDTA. The phage protein coats were degraded using proteinase K (1 mg/ml) and SDS (1%). A phenol-chloroform extraction was performed, and DNA was precipitated with ethanol. The washed DNA pellet was resuspended in T_10 _E_1 _buffer (10 mM Tris-HCl (pH 8.0), 1 mM EDTA) and stored at -20°C.

### Shotgun library construction and sequencing

Shotgun subclone libraries were constructed at Lucigen Corporation (Middleton, WI) as previously described [35]. Briefly, phage genomic DNA was randomly sheared using a Hydroshear instrument (Digilab Genomic Solutions, Ann Arbor, MI) and DNA fragments from 1 to 3 kb in size were extracted from an agarose gel. Phage DNA fragments were blunt-end repaired, ligated to asymmetric adapters, amplified using a proof reading polymerase and ligated into the pSMART^® ^GC cloning vector following manufacturer recommendations. The ligation was transfected into electrocompetent *E. coli *cells. *E. coli *transformants were robotically picked into Luria-Bertani (LB) broth containing 30 ug per ml kanamycin and 10% (w/v) glycerol in a 96-well format using a QPix2 colony picking system (Genitex Limited, Hampshire, UK). Colony PCR was performed on a representative number of clones (n = 10) to assess insert size and the percentage of subclones containing an insert. Plasmid DNA was isolated using standard alkaline-SDS lysis and ethanol precipitation. Alternately, the insert was amplified from the *E. coli *clone glycerol stock using a pSMART vector-specific primer set, with 30 cycles of amplification (95°C denaturation, 50°C annealing, and 72°C extension). The resultant PCR products were treated with exonuclease I and Shrimp Alkaline Phosphatase to remove oligonucleotides. Sanger sequencing from both ends of the insert was obtained using ABI PRISM BigDye™ 3.1 Terminators chemistry (Applied Biosystems, Foster City, CA), and sequencing products were resolved on an ABI 3130XL capillary electrophoresis instrument.

### Contig assembly and primer walking

Raw sequence data from eiMSLS was re-assembled using LaserGene software (DNASTAR Inc., Madison, WI). The eiMSLS sequence was used as a reference for alignment of eiAU and eiDWF sequences. For the latter two genomes, raw sequence data was trimmed for quality and vector sequence was removed using Sequencher™ software (Gene Codes Corporation, Ann Arbor, MI). Contigs were re-assembled using CromasPro v.1.42 (Technelysium Pty, Tewantin, Australia) using 70% sequence match, and a minimum of 30 bp overlap. Contigs were manually edited to remove nucleotide gaps and mis-called bases. Closure of each respective phage genome was completed by primer walking using either the isolate phage DNA or amplified products as the sequencing template. Each phage was determined to have a circular genome by PCR amplification using primers directed out from the ends of the single large contig comprising the respective phage genome.

### Genome sequence analysis

Open reading frames were identified using a GeneMark heuristic approach for gene prediction in prokaryotes, which is specifically designed for small virus, plasmid, or phage genomes less than 50 kb in size [36]. Additionally, GLIMMER 3.02, and NCBI's ORF Finder [37] were utilized to corroborate the predicted ORFs obtained from GenMark analysis. The % GC content of phages was calculated using geecee [38]. The tRNAscan-SE v.1.21 program was used to search for tRNA genes [39]; [40]. Gene function was predicted by comparing each phage ORF sequence against the GenBank nr/nt sequence database using the BLASTp and BLASTn [41] search algorithms. Iterative PSI-BLAST analysis was used to increase sensitivity of detecting homologous genes for ORFs resulting in hits with low E-values. Searches for secondary structures (profiles, patterns, blocks, motifs, and protein families) were performed using a web server [42]. Frameshifts were detected using FrameD [43]. The amino acid identity (AAI) of predicted protein sequences was determined by pairwise BLASTp analysis of each set of phage homologs. Dotplots were generated using the DOTMATCHER tool from EMBOSS (Ian Longden, Sanger Institute, Cambridge, UK). Pairwise global alignment and graphical representation of phage genomes was performed using the CGView server using tBLASTx with an E-value cutoff of 0.001 [44]. Genome sequences were annotated using the Artemis software package (The Institute for Genomic Research), and all sequences were deposited in the GenBank database (accession # HQ824548-HQ824705) using Sequin.

### Phylogenetic analysis

The predicted amino acid sequences for phage terminase large subunit and DNA polymerase were used to conduct a phylogenetic analysis of these *E. ictaluri *bacteriophages. The amino acid sequence for each predicted protein was aligned with a collection of homologous sequences using the program ClustalW2 [45]. ClustalW2 multiple alignments were exported to Mega4 [46] and a maximum parsimony analysis was used to construct a phylogenetic tree, with bootstrap support (n = 1000 replicates).

## Competing interests

The authors declare that they have no competing interests.

## Authors' contributions

AC prepared phage DNA, assembled and finished phage genomes, conducted the phage genome annotation and phylogenetic analyses, and drafted the manuscript. TJW isolated the eiMSLS phage, prepared phage DNA, and contributed to finishing the eiMSLS genome sequence. GCW produced the primary DNA sequence for all three phage genomes, and assembled and finished one phage genome. DAM prepared phage genome shotgun subclone libraries for sequencing. JST was the primary supervisor of AC, helped coordinate the collaborative work, and contributed to the intellectual design of the project. MRL co-supervised AC, helped in the assembly and finishing of the phage genomes, and in the manuscript design and editing. All authors read and approved the final version of the manuscript.
